# Global trends in the burden of alcohol use disorders in the working-age population from 1990 to 2021 and projections for the next 20 years

**DOI:** 10.3389/fpubh.2025.1616343

**Published:** 2025-07-28

**Authors:** Xinyu Cui, Kexin Liu, Yuanyi Ji, Su Han, Yongzhong Cheng

**Affiliations:** ^1^West China School of Public Health and West China Fourth Hospital, Sichuan University, Chengdu, China; ^2^West China Hospital, Sichuan University, Chengdu, China

**Keywords:** global burden of disease, projections, alcohol use disorders, working-age population, global trends

## Abstract

**Introduction:**

Alcohol use disorders (AUD) have long been among the most disabling mental disorders and a leading cause of health loss. However, data on the burden and trends of these disorders among the working-age population are scarce.

**Methods:**

This study aimed to assess trends in the burden of AUD among people aged 15-64 years from 1990 to 2021 at the global, regional and national levels and to project future trends. Based on the trend analysis of the Global Burden of Disease Study 2021 (GBD2021), we report age-standardized data and estimated annual percentage changes (EAPC) for the incidence, prevalence, mortality, and disability-adjusted life years (DALYs) of AUD in the working-age population at the global, regional, and national levels and analyze global trends by age, sex, and social development index (SDI). Furthermore, critical inflection points and local trends Average Annual Percent Change (AAPC) were further explored via joinpoint regression analysis, and the burden of AUD was predicted via the Bayesian age-period-cohort (BAPC) model.

**Results:**

Globally, the incidence number of AUD cases among people aged 15-64 years in 2021 was estimated to be 51340.37 × 10^3^ (95%UI 37577.93 × 10^3^ −68135.93 × 10^3^). During the period from 1990 to 2021,the age-standardized incidence rate (ASIR) (EAPC = −0.85, 95%CI: −0.89 to −0.81, *p* < 0.05), age-standardized mortality rate(ASMR) (EAPC = −1.98, 95%CI: −2.43 to −1.54, *p* < 0.05) and age-standardized disability-adjusted life year (DALY) rate (ASDR) (EAPC = −1.39, 95%CI: −1.59 to −1.19, *p* < 0.05) decreased significantly. In terms of regions, from 1990 to 2021, the disease burden in areas with a medium--to-high socio-demographic index (SDI) was greater, and this trend was particularly pronounced in Eastern Europe.Disease burden and SDI level showed a trend of stage correlation, ASDR (*R* = 0.28, *p* < 0.001). At the national level, the country with the highest disease burden was Mongolia,the prevalence rate in 2021 will be 7087.13 cases per 100,000 (95% UI:5192.08 to 9339.88), while Mongolia leads in terms of DALYs and mortality rates. It is estimated that by 2044, the global ASPR and ASDR will reach 766.67/100,000 and 205.88/100,000, respectively.

**Discussion:**

Despite the decline in AUD among the working population over the past 30 years, significant differences remain between genders,regions and ages, and these differences continue to have important public health consequences. In the face of diverse interests and the reality of global health inequalities, strategies to prevent and reduce the burden of disease still require sustained efforts. Over the past 30 years, AUD have seen a notable decline. The passage of the 2022-2030 Global Alcohol Action Plan marks a pivotal moment in global policy formulation. Despite the various interests and the reality of global health inequalities, these disparities continue to yield significant public health consequences. Efforts to minimize the health losses caused by alcohol consumption and prioritize interventions targeting labor populations are particularly important.

## 1 Introduction

Alcohol use disorders (AUD) include diseases characterized by compulsive heavy drinking and loss of control over alcohol intake (1). People with such disorders have impaired control over their alcohol consumption and chronically exhibit heavy and often continuous use of alcohol (2, 3). To date, alcohol use has been identified as one of the top ten major risk factors for disease burden in all global comparative risk assessments (4). Globally, AUD represent the most prevalent form of all substance use disorders (5). In 2016, an estimated 3 million [95% uncertainty interval (UI): 2.6–3.6] alcohol-attributable deaths and 131.4 million (95% UI: 119.4–154.4) disability-adjusted life-years (DALYs) were reported, accounting for 5.3% (95% UI: 4.6–6.3) of all deaths and 5.0% (95% UI: 4.6–5.9) of all DALYs worldwide (6, 7). The alcohol-attributable burden of disease was higher among men than among women,at the same time there are signs that this gender gap is narrowing over time (8). In addition, the prevalence of AUD was highest in both high-income countries (8.4, 95%CI 8.0–8.9) and upper-middle-income countries (5.4, 95%CI 5.0–6.0) for both sexes (9). Furthermore, AUD are also influential factors in the burden of other diseases (10). Persistent heavy drinking not only damages the cardiovascular, gastrointestinal and immune systems but also increases the risk of heart disease, stroke and cirrhosis (11, 12). In addition, there are serious harmful costs not only for personal health but also for family, friends and society as a whole. Despite their important public health consequences, AUD remain among the most undertreated mental disorders (13, 14). In a large study of more than 13,000 patients and 358 general practitioner representatives in six European countries, only 22.3% of alcohol-dependent patients received interventions (15).

Previous studies have documented regional disparities in the disease burden of AUD across the general population; however, research specifically targeting labor populations remains limited (16). According to international standards, the labor population typically refers to individuals aged 15 to 64 years, who are crucial contributors to socio-economic development and play a vital role in societal progress (17). This demographic faces significant psychosocial stress in both personal and professional life (18). Notably, the majority of individuals engaging in harmful alcohol consumption worldwide are young people, primarily young males. AUD severely impair individual health, exacerbate the risk of chronic diseases and mental health issues, and result in premature deaths that are largely preventable. Furthermore, such disorders impose a substantial burden on families and communities by increasing the likelihood of accidents, injuries, and violence (1). Given the current lack of comprehensive analysis on AUD among this age group, this study aims to assess the current situation and conduct predictive analyses. These findings will provide valuable insights for developing targeted prevention and treatment strategies.

This study aims to (1) describe the disease burden of AUD among the labor population at the global, regional, and national levels; (2) quantify the time trend changes and key years of AUD at the global and regional levels from 1990 to 2021; and (3) predict the age-standardized prevalence rates and DALYs of AUD globally for the next 20 years. By identifying and analyzing high-risk groups, this study will contribute to understanding the scope and impact of the issue, thereby providing valuable insights for developing effective social support and intervention measures, including public health education and health promotion, community interventions, tax policies, and restrictions on alcohol sales and marketing. These efforts are crucial for improving public health, reducing economic losses, promoting social welfare, and advancing health equity.

## 2 Materials and methods

### 2.1 Data sources

This study is based on secondary analysis of data from the Global Burden of Disease Study 2021 (GBD2021), which was released on May 16, 2024. The study utilized data from various sources, including government websites, statistical yearbooks, vital registration systems, ESRD registries, household surveys, hospital records, disease and population registries, and published literature, to ensure the quality and breadth of the analyses (19, 20). The study covered 204 countries and territories, providing age- and sex-specific quantifications of 371 diseases and injuries, as well as 88 risk factors. It conducted detailed estimates by age, sex, location, and year. The GBD study framework was used to standardize and ensure comparability across different populations and time points (21, 22). Similar methods to previously published studies were adopted, and comprehensive GBD results were obtained using a query tool (https://vizhub.healthdata.org/gbd-results/). We extracted data on the incidence rate, prevalence, deaths, and disability-adjusted life-years (DALYs) (one DALY represents the loss of one full year of healthy life due to premature death or disability) and their 95% uncertainty intervals (UIs) for the population aged 15–64 years globally from 1990 to 2021. Additionally, data on age groups, population, and the Socio-demographic Index (SDI) were included for comprehensive analysis. To eliminate the impact of population age structures on overall rates and enable comparisons of disease burden across different years and regions, age standardization was performed to obtain age-standardized rates (ASR).

### 2.2 Definition and classification of AUD

Alcohol Use Disorder (AUD) is a chronic disease resulting from long-term alcohol consumption, characterized by physical and psychological dependence on alcohol, as well as impairment in social functioning. For related definitions, refer to DSM-5, ICD-10, or ICD-11. AUD is closely associated with patterns of long-term heavy drinking, and some scholars suggest that this specific type of drinking behavior should be incorporated into the definition of AUD (23–25).

### 2.3 Descriptive analysis

To comprehensively understand the disease burden of AUD, descriptive analyses were conducted at the global level, across 21 GBD regions, and in 204 countries/territories. First, we compared the AUD incidence rate, prevalence, mortality, and DALYs (both in terms of cases and age-standardized rates, ASR) between 1990 and 2021, as well as between males and females. Second, we analyzed age-related changes in ASR for AUD burden across five Socio-demographic Index (SDI) levels. Additionally, we examined trends at the SDI and regional levels and their correlations. Finally, we compared the AUD incidence rate, mortality, and DALY burden in 2021 and conducted a visualization of these data on a world map.

### 2.4 SDI analysis

The SDI serves as a comprehensive indicator for assessing the socio-economic status of countries and regions. A higher score indicates a stronger socio-economic development. This index is derived from an integrated assessment of data such as the total fertility rate among women under the age of 25, the average educational attainment of women aged 15 and above, and per capita income. The hypothetical values of SDI range from 0 to 1. According to the classification standards of GBD, countries and regions are divided into five quintiles: high SDI (>0.81), high-middle SDI (0.70–0.81), middle SDI (0.61–0.69), low-middle SDI (0.46–0.60), and low SDI (<0.46). To analyze the correlation between SDI and the burden of AUD, we utilized the geom_smooth function from the ggplot2 package, applying a locally estimated scatterplot smoothing (loess) model. This analysis covered data from 21 global regions, aiming to evaluate the relationship between them.

### 2.5 Trend analysis

The joinpoint regression models are a collection of linear statistical models used to assess trends in the burden of AUD over time. The calculation method for this model involves using the least squares method to estimate the pattern of disease rate changes, thereby avoiding the subjectivity that can occur in typical trend analyses based on linear trends. By calculating the sum of the squares of the residuals between the estimated and actual values, the turning points of the trend can be determined. The Joinpoint software (version 4.9.1.0, developed by the National Cancer Institute in Rockville, Maryland, United States) is employed to create this model. The specific operations involve using the command-line-based Joinpoint software to perform Joinpoint regression analysis, with time specified as the independent variable and incidence rate, prevalence, mortality rate, and age-standardized DALY rates as the dependent variables. We also calculated the Average Annual Percentage Change (AAPC) and compared it to zero to investigate whether the fluctuations in different segments were statistically significant.

### 2.6 Predictive analysis

To more accurately predict and explore the future burden of AUD over the next 20 years, we employed the Bayesian Age-Period-Cohort (BAPC) analysis model and Integrated Nested Laplace Approximation (INLA) to forecast the global burden in 2044 (26). This model has unique advantages in that it can account for age, period, and cohort effects to accurately estimate future disease burden (27). The BAPC model utilizes INLA to estimate marginal posterior distributions, resolving issues related to mixing and convergence that are often encountered with Markov Chain Monte Carlo (MCMC) methods used in traditional Bayesian approaches. The model effectively handles age-stratified incidence and mortality rates, making it particularly valuable for predicting future trends in scenarios where significant changes in population structure are expected (28). The advantage of the BAPC model lies in INLA’s ability to efficiently approximate marginal posterior distributions, avoiding the problems associated with traditional MCMC sampling. Due to its strengths, this model is well-suited for long-term disease burden predictions and has been widely applied and recognized (29). In particular, it has been extensively used in GBD studies involving changes in age composition and complex cohort effects (30).

### 2.7 Statistical analysis

Our study strictly adhered to the Guidelines for Accurate and Transparent Health Estimates Reporting (GATHER). This study does not require ethical approval and informed consent as it utilized the GBD database, an open dataset containing anonymous participant data. Throughout the analysis, statistical significance was determined at a *p*-value threshold of <0.05. For all computations and analyses, we utilized the R software (version 4.4.1) to perform the database construction, collation, statistical analysis and visualization.

## 3 Results

### 3.1 Global trends

Globally, the number of incident AUD cases among individuals aged 15–64 years in 2021 was estimated to be 51,340.37 × 10^3^ (95% UI 37,577.93 × 10^3^–68,135.93 × 10^3^), representing a 27.73% increase. The age-standardized incidence rate of AUD in 2021 for the 15–64 age group was 997.17 (95% UI 728.64–1,325.41) per 100,000 population, which was lower than the 1990 value of 1,252.43 (95% UI 898.69–1,681.99). Additionally, the age-standardized incidence rate of AUD in the global working-age population decreased by an average of −0.85% (95% CI -0.89 to −0.81) per year between 1990 and 2021.

In 1990, the number of deaths due to AUD in the working-age population aged 15–64 years was estimated to be 95.46 × 10^3^ (95% CI 88.13 × 10^3^–100.37 × 10^3^), while in 2021, it increased to 125.68 × 10^3^ (95% CI 101.95 × 10^3^–139.56 × 10^3^), representing a 31.66% rise. After age standardization, the age-standardized mortality rate was 3.30 per 100,000 (95% UI 3.05–3.47) in 1990 and 2.35 per 100,000 (95% UI 1.90–2.61) in 2021, with a global average annual decline of −1.98% (95% CI -2.43 to −1.54) over the 30-year period.

In 2021, the disability-adjusted life years (DALYs) due to AUD among the global working population were estimated to be 15,282.14 × 10^3^ (95% UI 11,562.32 × 10^3^–20,343.15 × 10^3^), and the age-standardized DALY rate per 100,000 population was 293.13 (95% UI 221.15–391.48). From 1990 to 2021, the age-standardized DALY rate for AUD decreased, with a global average annual change of −1.39% (95% CI -1.59 to −1.19) (Table 1).

**Table 1:** Numbers and ASRs of AUD burden and their temporal trend from 1990 to 2019.

Year	Both	Male	Female
1990			
Incidence/1000 (95%Ul)	40195.07 (28725.61–54215.18)	31349.82 (22603.01–41914.21)	8845.25 (6093.82–12312.23)
Deaths/1000 (95% UI)	95.46 (88.13–100.37)	80.94 (73.47–85.71)	14.52 (13.80–15.12)
Prevalence/1000 (95% UI)	79081.53 (58658.04–103976.22)	61607.02 (46037.19–80195.45)	17474.51 (12502.19–23763.84)
DALYs/1000 (95% UI)	12185.69 (9197.00–16190.08)	9821.74 (7482.35–12913.28)	2363.96 (1692.50–3286.15)
ASIR/100,000persons (95%Ul)	1252.43 (898.69–1681.99)	1933.86 (1399.74–2575.24)	554.97 (383.92–769.27)
ASMR/100,000persons (95%UI)	3.30 (3.05–3.47)	5.53 (5.03–5.86)	1.02 (0.97–1.06)
ASPR/100,000persons (95%UI)	2491.48 (1855.67–3259.75)	3849.55 (2888.39–4987.26)	1102.95 (792.86–1492.66)
ADSR/100,000persons (95%UI)	392.80 (298.90–516.97)	627.97 (482.13–818.07)	152.29 (110.17–209.51)
2021			
Incidence/1000 (95%Ul)	51340.37 (37577.93–68135.93)	40360.43 (29868.74–53010.19)	10979.94 (7590.97–15272.15)
Deaths/1000 (95%UI)	125.68 (101.95–139.56)	108.95 (85.48–122.56)	16.73 (15.83–17.85)
Prevalence/1000 (95% UI)	99569.51 (75171.28–129067.77)	77890.41 (59400.47–99966.09)	21679.10 (15588.56–29375.50)
DALYs/1000 (95% UI)	15282.14 (11562.32–20343.15)	12452.06 (9487.91–16457.58)	2830.09 (2009.21–3953.21)
ASIR/100,000 persons (95%Ul)	997.17 (728.64–1325.41)	1556.49 (1150.31–2046.65)	431.54 (297.67–601.54)
ASMR/100,000 persons (95%UI)	2.35 (1.90–2.61)	4.08 (3.20–4.59)	0.62 (0.59–0.66)
ASPR/100,000persons (95%UI)	1925.07 (1450.57–2499.91)	2992.56 (2278.53–3846.04)	848.36 (608.74–1152.06)
ADSR/100,000persons (95%UI)	293.13 (221.15–391.48)	475.37 (361.42–629.89)	109.80 (77.57–154.09)
1990–2021			
Incidence (%)	27.73	28.74	24.13
Deaths (%)	31.66	34.61	15.22
Prevalence (%)	25.91	26.43	24.06
DALYs (%)	25.41	26.78	19.72
EAPC of ASIR (95% CI)	-0.85 (−0.89 to −0.81)	−0.84 (−0.88 to −0.8)	−0.82 (−0.91 to −0.73)
EAPC Of ASMR (95% CI)	−1.98 (−2.43 to −1.54)	−1.8 (−2.22 to −1.38)	−2.8 (−3.38 to −2.21)
EAPC Of ASPR (95% CI)	−0.96 (−1 to −0.92)	−0.97 (−1.01 to −0.92)	−0.85 (−0.95 to −0.76)
EAPC of ASDR (95% CI)	−1.39 (−1.59 to −1.19)	−1.35 (−1.54 to −1.15)	−1.45 (−1.69 to −1.22)

ASIR, age-standardized incidence rate; ASMR, age-standardized mortality rate; ASPR age-standardized Prevalence rate; ASDR, age-standardized disability-adjusted life years rate; EAPC, estimated annual percentage change; UI, uncertainty interval; CI, confidence interval.

### 3.2 Gender and age differences

In 2021, the number of males aged 15–64 years in the working population with AUD was estimated to be 40,360.43 × 10^3^ (95% UI 29,868.74 × 10^3^–53,010.19 × 10^3^), which was 3.68 times higher than that of females during the same period. From 1990 to 2021, the average annual incidence of AUD among males and females worldwide showed distinct trends. For males, the incidence rate decreased by −0.84 (95% CI -0.88 to −0.80), from 1,933.86 per 100,000 in 1990 (95% UI 1,399.74–2,575.24) to 1,556.49 per 100,000 in 2021 (95% UI 1,150.31–2,046.65). For females, the incidence rate decreased by −0.82 (95% CI -0.91 to −0.73), from 554.97 per 100,000 in 1990 (95% UI 383.92–769.27) to 431.54 per 100,000 in 2021 (95% UI 297.67–601.54).

The age-standardized Prevalence rate of AUD among men decreased from 3,849.55 per 100,000 in 1990 (95% UI 2,888.39–4,987.26) to 2,992.56 per 100,000 in 2021 (95% UI 2,278.53–3,846.04), with an average annual decline of −0.97 (95% CI -1.01 to −0.92). For women, the ASPR decreased from 1,102.95 per 100,000 in 1990 (95% UI 792.86–1,492.66) to 848.36 per 100,000 in 2021 (95% UI 608.74–1,152.06), with an average annual decline of −0.85 (95% CI -0.95 to −0.76).

Over the past 30 years, the annual age-standardized mortality rate declined more steeply for women (−2.8, 95% CI -3.38 to −2.21) compared to men (−1.8, 95% CI -2.22 to −1.38). Similarly, the annual decline in disability-adjusted life years (DALYs) was greater for women (−1.45, 95% CI -1.69 to −1.22) than for men (−1.35, 95% CI -1.54 to −1.15) (Table 1).

**Figure 1:**
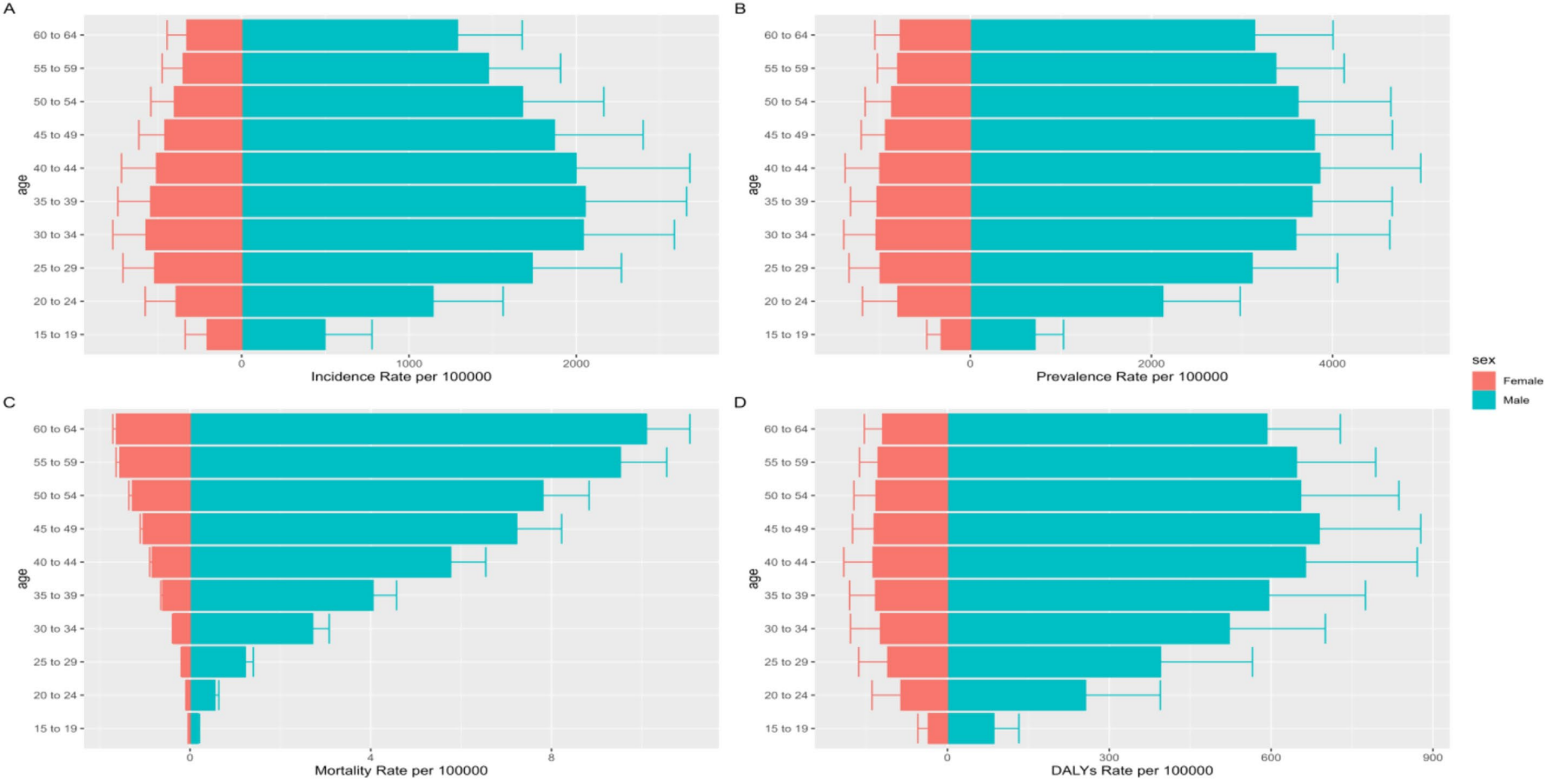
15–64 Years old global incidence rate **(A)**, Prevalence rate **(B)**, Mortality rates **(C)**, DALYS rate **(D)** of alcohol use disorders (AUD), 2021.

In terms of age distribution among the working population, in 2021, both men and women exhibited the highest incidence rates in the 30–34 and 35–39 age groups, consistent with the highest incidence rates in 1990. For prevalence, men aged 40–44 years had the highest burden, whereas women in the relatively younger 30–34 age group were most affected, differing from the 1990 data, which showed the highest burden in the 35–39 age group for men. The mortality rate among the working population in 2021 increased with age, differing from 1990, when the highest burden was observed in the 50–59 age group. Finally, in 2021, the burden of DALYs was highest among men aged 45–49 years and women aged 40–44 years, reflecting a shift toward younger ages compared to the disease burden 30 years ago, which was highest among those aged 50–54 years (Figure 1).

### 3.3 Local trends in AUD using joinpoint analysis

**Figure 2:**
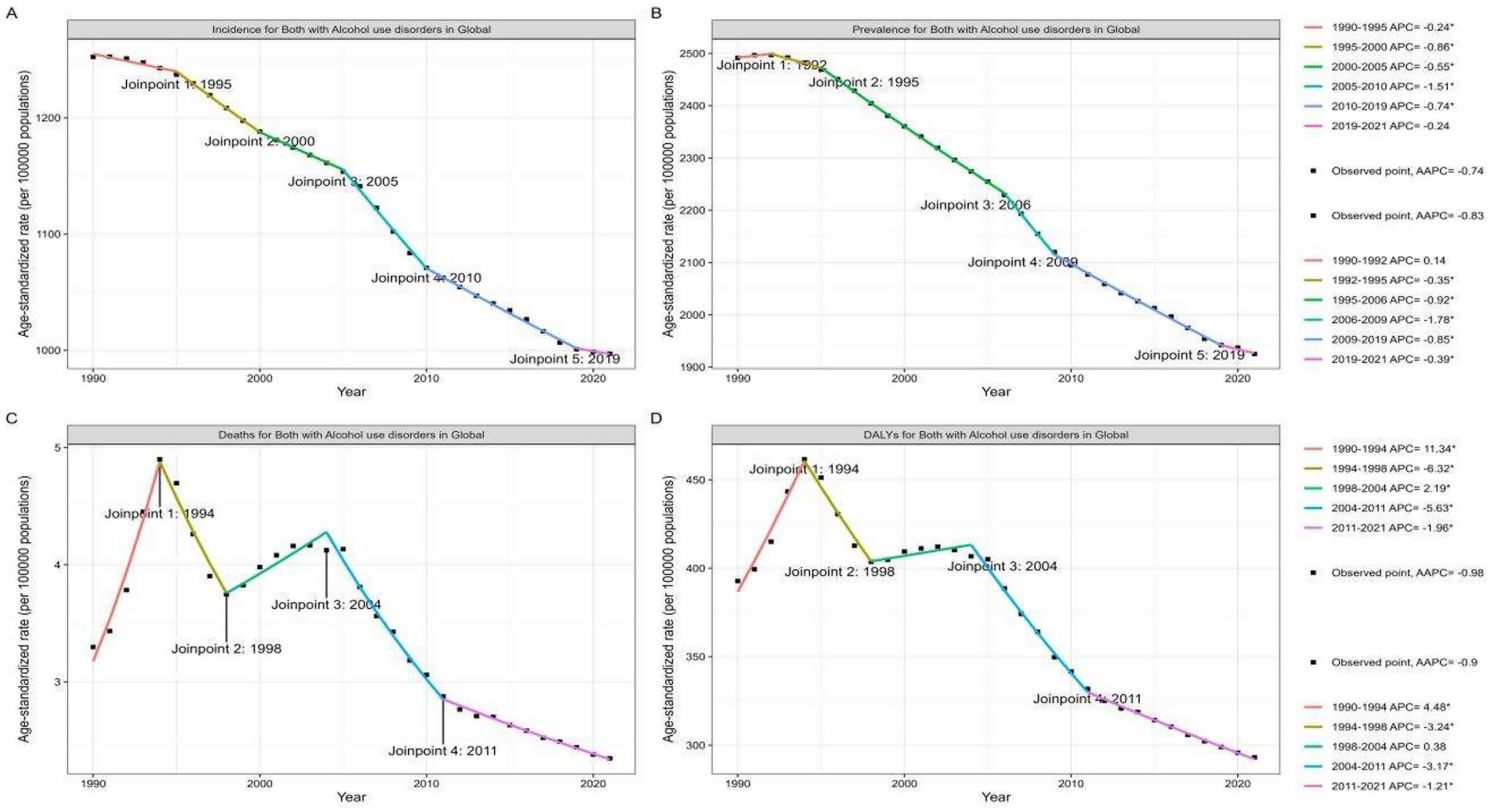
The join point regression analysis of age-standardized rate of AUD incidence **(A)**, prevalence **(B)**, mortality **(C)**, and DALYs **(D)**, in labor force aged 15–64 years from 1990 to 2021. DALYs, disability-adjusted life years; APC, annual percentage change; AAPC, average annual percent change.

To further explore the local changes in the above indicators on a global scale, joinpoint analysis revealed that the incidence of AUD exhibited a gradual downward trend starting from 1995, 2000, 2005, 2010, and 2019 (AAPC = −0.74). Notably, 1992 marked a critical inflection point following an increase in the prevalence of AUD, while 1995, 2006, 2009, and 2019 were identified as key turning points where a substantial decline became evident (AAPC = −0.83). Furthermore, the model highlighted that 1994, 1998, 2004, and 2011 were significant turning points for both mortality (AAPC = −0.98) and disability-adjusted life years (AAPC = −0.9). Specifically, both indicators shifted from a decline to an increase in 1998, while 1994 and 2004 marked the transition from an increasing to a decreasing trend. This was followed by a significant decline until 2011 and a continued decrease thereafter (Figure 2).

### 3.4 SDI and 21 regional AUD trend analysis

We found that in terms of age-standardized incidence, prevalence, DALYs and mortality, all regions with the highest burden of AUD are located in high SDI regions and medium–high-SDI regions, followed by medium–low-SDI regions and medium-SDI regions. The burden of disease shows a phased correlation with the SDI. In 2021, in low-SDI areas, the age-standardized incidence, mortality, and DALYs were 861.59/100,000 (95% UI 621.30–1,154.56), 1.63/100,000 (95% UI 1.04–2.14), and 229.97 per 100,000 (95% UI 164.33–316.85), whereas in areas with high-SDI, these indicators were 1,378.76 per 100,000 (95% UI 1,033.35–1,796.89), 3.27 per 100,000 (95% UI 3.15–3.39), and 409.16 (95% UI 313.33–543.53). The incidence rate was 1.60 times that of the low-SDI area, the mortality rate was 2 times that of the low-SDI area, and the DALYs were 1.78 times that of the low-SDI area. Fortunately, all of the above indicators showed a downward trend year by year, especially in the High-middle SDI areas, with age-standardized mortality, and DALYs decreasing by an average of −3.54 (95% CI -4.44 to −2.62) and −2.4 (95% CI -2.9 to −1.89) per year, respectively. (Table 2).

**Table 2:** Labor force aged 15–64 EAPC of ASIR, ASMR, ASPR and ASDR for AUD in countries with five SDl levels from 1990 to 2021.

Region	ASIR/100,000 persons (95% Ul) (1990/2021)	EAPC of ASIR (95% CI)	ASMR/100,000 persons (95% Ul) (1990/2021)	EAPC of ASMR (95% CI)	ASPR/100,000 persons (95% Ul) (1990/2021)	EAPC of ASPR (95% CI)	ASDR/100,000 persons (95%UI)(1990/2021)	EAPC of ASDR (95% CI)
Low SDI	1009.58 (701.90–1385.75)/861.59 (621.30–1154.56)	−0.47 (−0.49 to −0.45)	1.96 (1.36–2.41)/1.63 (1.04–2.14)	−0.75 (−0.81 to −0.68)	1926.05 (1383.10–2585.15)/ 1630.69(1208.64–2137.77)	−0.49 (−0.52 to −0.46)	272.04(191.81–372.97)/229.97(164.33–316.85)	−0.54 (−0.57 to −0.52)
Low-middle SDI	1167.17 (817.13–1597.29)/896.92 (649.33–1197.03)	−0.95 (−1.07 to −0.83)	2.89 (1.97–3.47)/2.35 (1.54–2.96)	−0.7 (−0.79 to −0.62)	2221.93 (1607.66–2977.46)/1699.13 (1269.86–2217.85)	−0.97 (−1.1 to −0.85)	348.42 (254.28–462.93)/271.95 (201.56–364.10)	−0.88 (−0.99 to −0.76)
Middle SDI	1047.30(731.42–1431.97)/879.25 (627.36–1188.05)	−0.82 (−0.9 to −0.74)	2.11(1.85–2.31)/1.56 (1.01–1.86)	−1.04 (−1.24 to −0.83)	2012.38 (1457.80–2697.23)/1676.82 (1232.59–2212.49)	−0.85 (−0.93 to −0.77)	296.39(216.53–402.53)/237.18(171.63–326.39)	−0.92(−0.99 to −0.85)
High-middle SDI	1480.13 (1077.40–1962.31)/1185.48 (869.31–1575.46)	−0.96 (−1.05 to −0.87)	5.43 (5.27–5.67)/3.20(2.92–3.49)	−3.54 (−4.44 to −2.62)	3087.03 (2334.38–3960.17)/2308.80(1744.32–2990.71)	−1.27 (−1.38 to −1.17)	547.24 (434.61–696.97)/370.88(286.38–487.41)	−2.4 (−2.9 to −1.89)
High SDI	1560.50 (1150.52–2064.30)/1378.76 (1033.35–1796.89)	−0.31 (−0.37 to −0.25)	3.40 (3.30–3.50)/3.27(3.15–3.39)	−0.27 (−0.37 to −0.16)	3166.72 (2414.12–4084.37)/2736.02 (2109.20–3484.60)	−0.36 (−0.42 to −0.31)	462.89 (348.16–621.04)/409.16 (313.33–543.53)	−0.38 (−0.43 to −0.33)

ASIR age-standardized incidence rate, ASMR age-standardized mortality rate, ASPR age-standardized Prevalence rate, ASDR age-standardized disability-adjusted life-year rate, EAPC estimated annual percentage change, AUD alcohol use disorders, SDI sociodemographic index, UI uncertainty interval, CI confidence interval.

**Figure 3:**
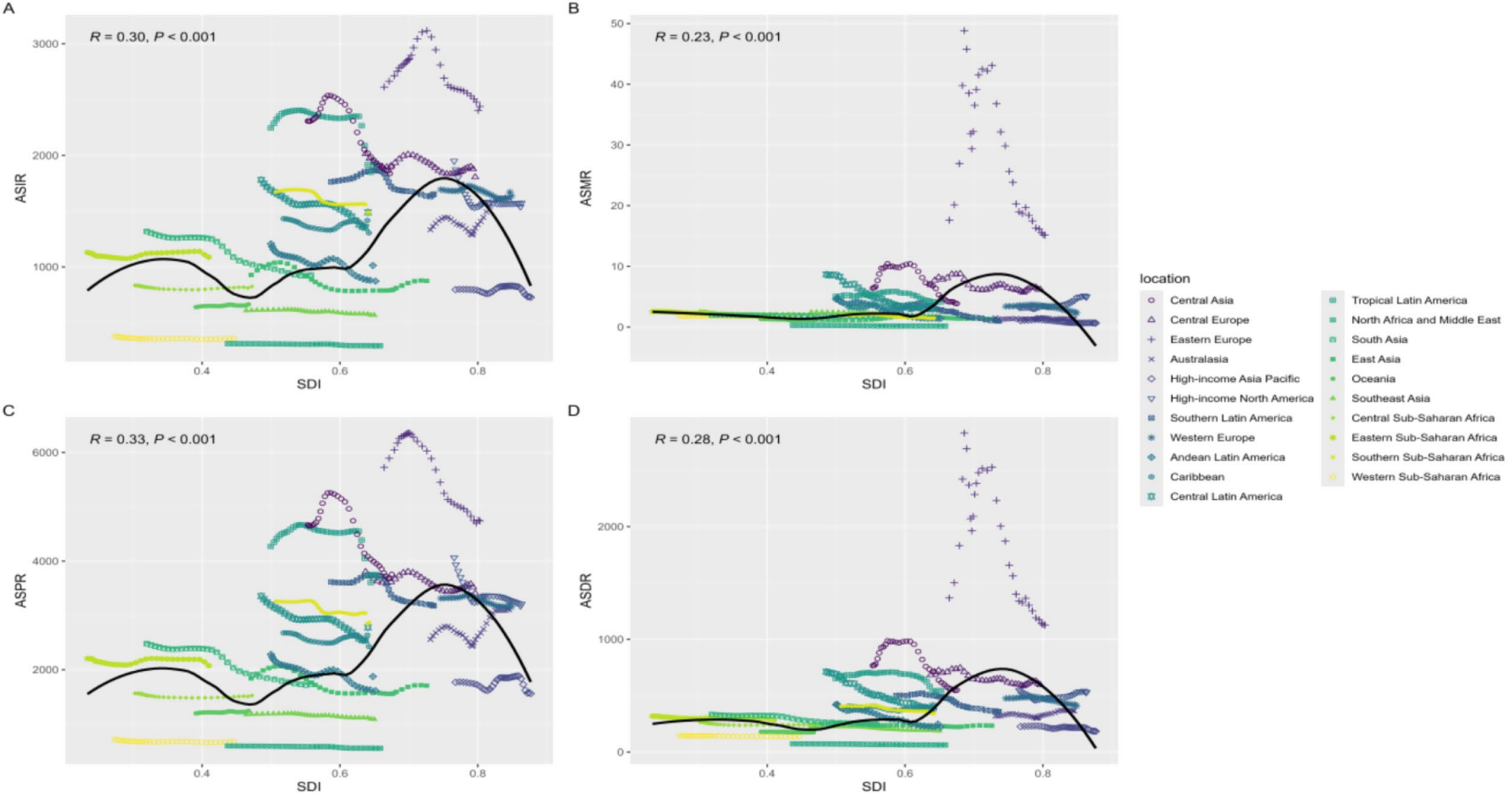
AUD for 21 GBD regions by sociodemographic index, 1990–2021; expected values based on Socio-demographic Index and disease rates in all locations are shown as the black line. **(A)** ASIR age-standardized incidence rate; **(B)** ASMR age-standardized mortality rate; **(C)** ASPR age-standardized Prevalence rate; **(D)** ASDR age-standardized disability-adjusted life-year rate. DALYs, disability-adjusted life years. Age-standardized DALYs rate for drug use disorders.

From 1990–2021, we further explored the relationships among AUD incidence, prevalence, mortality, and DALYs in regions with different SDI levels. The burden of disease is relatively high in regions with medium-to-high SDI levels and in high-SDI countries and relatively low in countries with medium-to-low and low SDI. We found that for the age-standardized incidence rate (ASIR) and age-standardized prevalence rate (ASPR), in regions with low SDI levels, there was a trend of first increasing and then decreasing with increasing SDI. The burden in East Sub-Saharan Africa and parts of South Asia is greater than expected (above the solid black line), whereas the burden in West Sub-Saharan Africa is much lower than expected (below the black solid line). In areas with medium–low SDI levels, there is a trend of first decreasing and then increasing with the SDI level, with AUD in the Middle East and North Africa and Southeast Asia being much lower than expected. In areas with medium SDI levels, the burden is greater in Central Asia and tropical Latin America, and in areas with medium–high SDI levels, Eastern Europe is much greater than expected. The ASDR and ASMR first increased but then decreased in the medium- and medium–high-SDI-level regions, with Eastern Europe and Central Asia having much higher values than expected. (Figure 3).

### 3.5 Country-level analysis

**Figure 4:**
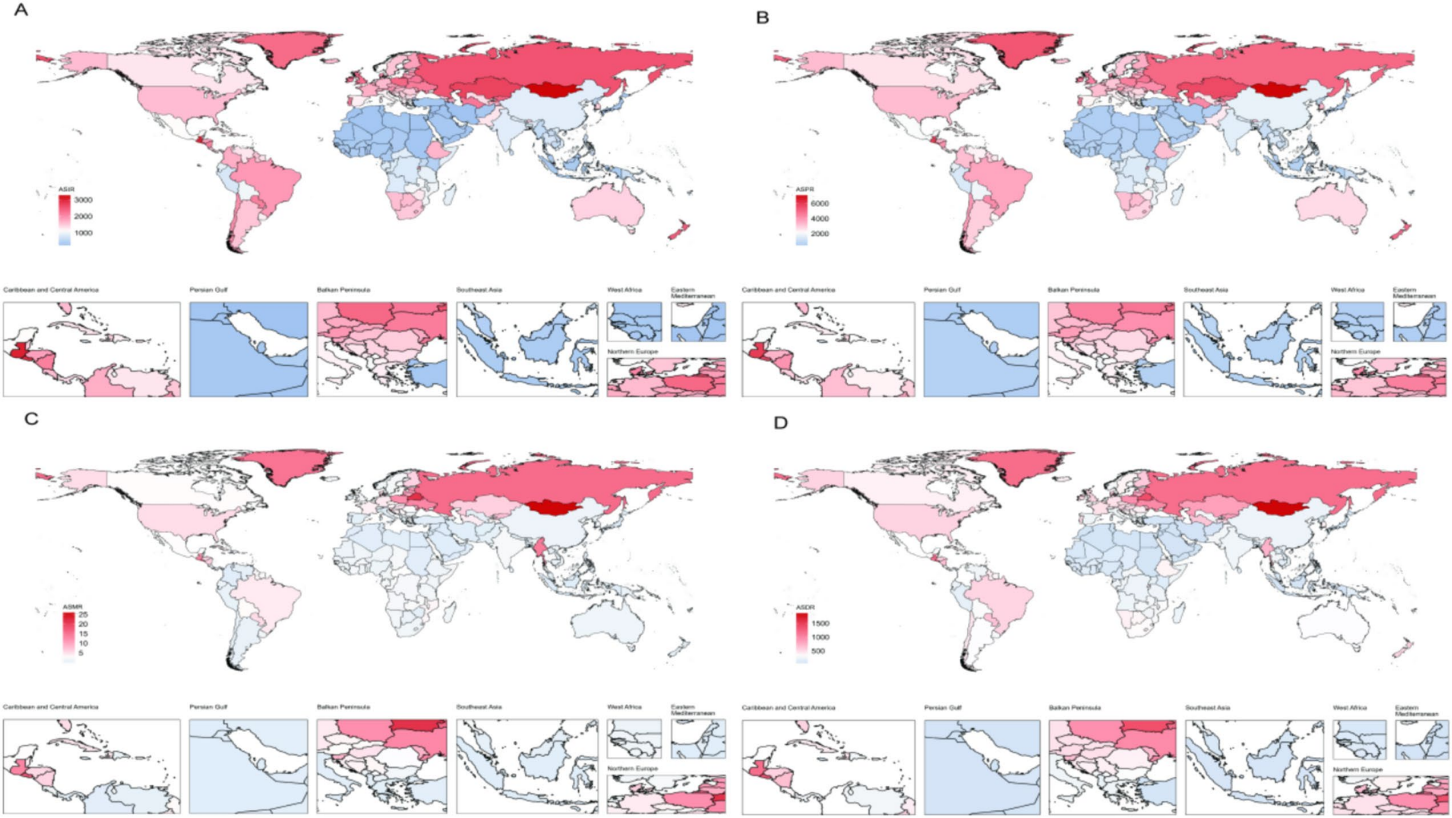
Burden of AUD in 2021 in 204 countries worldwide. ASIR **(A)**, ASPR **(B)**, ASMR **(C)**, and ASDR **(D)**.

In 2021, the age-standardized prevalence of AUD among people aged 15–64 worldwide was highest in Mongolia (3274.93/100,000; 95% UI 2381.20–4489.09), followed by Guatemala, El Salvador, Kazakhstan and the Russian Federation. In contrast, the Islamic Republic of Iran had the lowest prevalence (248.90 cases per 100,000 people; 95% UI 175.00–338.10). Subsequently, the Syrian Arab Republic, Egypt, Palestine, etc. Furthermore, Mongolia exhibited the highest prevalence rate, with 7087.13 cases per 100,000 population (95% UI 5192.08–9339.88), whereas the Islamic Republic of Iran demonstrated the lowest rate, with 473.55 cases per 100,000 population (95% UI 342.74–629.05). Additionally, Mongolia exhibited the highest rates of both DALYs and mortality, whereas Palestine demonstrated the lowest rate of DALYs. In general, the ranking of countries with high burdens at the national level was consistent for standardized incidence rates, prevalence rates, DALYs and mortality rates. (Figure 4).

### 3.6 Age-period-cohort analysis of AUD predicting the trend of age-standardized rates

**Figure 5:**
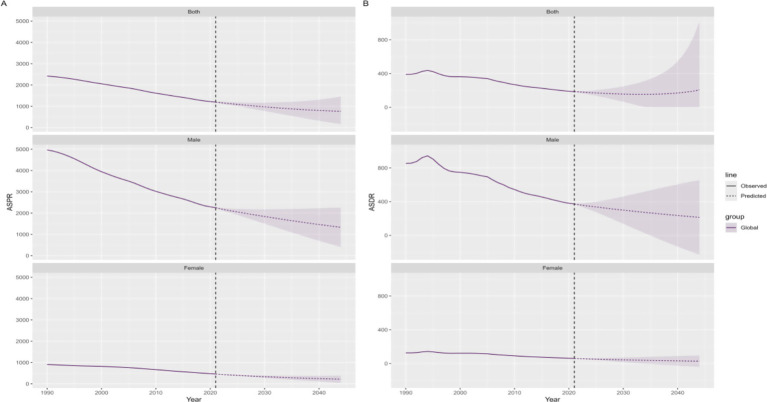
Trends in ASPR **(A)** and ASDR **(B)** from 2022 to 2044 predicted by Bayesian age-period-cohort (BAPC) models; ASPR, age-standardized prevalence rate; ASDR, age-standardized disability-adjusted life-year rate.

In order to predict the future global trend of the burden of disease in the working population by sex over the next twenty years, we conducted a Bayesian age-period-cohort (BAPC) model analysis. It is anticipated the age-standardized prevalence and DALY rates will demonstrate a persistent decline. It is estimated that by 2044, the age-standardized prevalence rate for the global working population will be approximately 766.67 cases per 100,000, with 1,332.76 cases per 100,000 for men and 217.63 cases per 100,000 for women, decrease of 60.20% compared to 2021; the age-standardized DALY rate for the global working population is estimated to be approximately 205.88 cases per 100,000, with 213.49 cases per 100,000 for men and 27.91 cases per 100,000 for women, decrease of 29.76% compared to 2021 (Figure 5).

## 4 Discussion

This study comprehensively considered factors such as gender, age, and SDI, and conducted a detailed epidemiological analysis of the disease burden trends of AUD among the working population from 1990 to 2021, while also forecasting future disease burden. Unlike previous studies that primarily focused on incidence or mortality rates in the general population, this analysis concentrated on the working population and employed age-standardized incidence rates, prevalence, mortality rates, and DALYs to measure disease burden. This approach provided new insights into the impact of AUD on a key population that plays a crucial role in societal and economic contributions (31–33). Over the past three decades, AUD exhibited a declining trend in terms of incidence, prevalence, mortality, and DALYs. Additionally, several key turning points were identified, which may be associated with changes in alcohol consumption patterns, alcohol availability, cultural and economic shifts, and alcohol marketing trends. These trends also align with global strategies, such as the World Health Organization’s (WHO) 2010 Global Strategy to Reduce Harmful Use of Alcohol and the WHO SAFER Initiative and Best Buys Report (34–37). These global strategies have provided guidance for countries to reduce harmful alcohol use through policy interventions (38). In terms of gender differences, men bore a significantly higher disease burden than women, although the gap in incidence and prevalence between genders has narrowed (16). However, reductions in mortality and DALYs were more pronounced among women. Therefore, targeted preventive interventions for men with AUD are essential to alleviate the disease burden, with men being a priority population for intervention (39). From the perspective of age distribution among the working population, the severity of DALYs demonstrated a trend toward younger age groups. Furthermore, as the population ages, the standardized mortality rate showed a positive correlation with age (38). These findings underscore the necessity of developing tailored guidelines to discourage alcohol consumption among young people and implementing alcohol control policies and interventions specifically targeting young males.

Previous studies have found that the burden of AUD is highest in regions with medium to high SDI, a finding consistent with our research results. As SDI increases, alcohol use and its harmful effects on health may pose growing challenges (2, 4, 40). Notably, the current disease burden in most low and low-to-middle SDI countries remains below that of high-to-middle SDI countries, a pattern associated with economic development and average alcohol consumption levels (41, 42). Central Asia, tropical Latin America, and certain Eastern European countries exhibit some of the highest AUD burdens globally, making them key regions for targeted preventive interventions. In contrast, the Middle East and North Africa report relatively lower disease burdens, likely due to cultural influences (33, 43). At the national level, Mongolia faces serious epidemiological challenges in addressing the burden of AUD and related problems. It has the highest levels of risky drinking behaviors among males, the rural population and those aged 25–34 years, driven by social, cultural and psychosocial factors (44, 45). The successful formulation and implementation of alcohol-related policies are complex processes influenced by numerous factors. However, non-policy factors also play a role in shaping alcohol consumption patterns and related harms (46). This study supports the development and implementation of alcohol control policies tailored to specific countries to further reduce alcohol-attributable disease burden in the near future. Finally, we predict that age-standardized prevalence and age-standardized DALYs will continue to decline over the next 20 years, with a two-fold reduction in men and nearly a four-fold reduction in women compared to 2021. Although the disease burden is decreasing, the health benefits gained from reductions in alcohol-attributable disease burden have not kept pace with overall health improvements globally. Therefore, alcohol remains a leading risk factor for disease burden worldwide, with its relative importance continuing to grow (16, 47).

Despite these findings, our study has several limitations that necessitate further investigation and resolution in future research. First, differences in data collection and reporting standards across countries and regions may compromise data accuracy, reliability, and comparability. The GBD database primarily aggregates data from national and regional reports and publications rather than direct national reporting. This approach may result in limitations regarding data completeness, timeliness, and quality, particularly in low-income settings where access to original data may be restricted, potentially hindering the accuracy of estimates by GBD researchers (43). Second, while the diagnostic criteria for AUD in this study were based on the well-established definitions provided by DSM-5 and ICD-10, the identification and diagnosis of early-stage cases may still be subject to underreporting, which could introduce bias into the data (23). Third, variations in data sources and methodologies may affect the accuracy of burden estimates. Future studies should prioritize the acquisition of higher-quality, standardized epidemiological data to improve the reliability and accuracy of global AUD disease burden assessments and predictions (48).

## 5 Conclusion

AUD remains a significant component of the global disease burden, not only in the past but also in the future, with profound implications for working populations, their families, and the overall socio-economic development. While the global burden has eased over the past few decades, notable disparities persist across gender, region, and age. Targeted interventions are urgently needed to adapt preventive and treatment strategies to meet specific demands. Public health interventions must account for these disparities to ensure that resources are allocated and implemented in a rational and precise manner (49). Furthermore, it is essential to strengthen global and regional collaboration, enhance the accessibility and availability of identification and intervention services, and promote the implementation of the “Global Action Plan for Healthy Lives and Well-being for All (2022–2030)” to reduce alcohol-related harms, decrease deaths and disabilities, and improve global health and well-being (50).

## Data Availability

Publicly available datasets were analyzed in this study. This data can be found at: https://vizhub.healthdata.org/gbd-results/.
